# Preserving Natural Bone With Bone Window Guided Endodontic Surgery Utilizing a Piezoelectric Device: Case Reports of Two Molars

**DOI:** 10.1155/crid/3731270

**Published:** 2026-07-29

**Authors:** Zaher Altaqi, Mohammed Mashyakhy

**Affiliations:** ^1^ Independent Researcher, Riyadh, Saudi Arabia; ^2^ Department of Restorative Dental Sciences, College of Dentistry, Jazan University, Jazan, Saudi Arabia, jazanu.edu.sa

**Keywords:** case report, guided endodontic surgery, piezoelectric device

## Abstract

**Aim:**

This study demonstrated the clinical application of static guided surgery (SGS) combined with piezoelectric technology for endodontic microsurgery. The research objective was to evaluate the benefits of integrating advanced technologies to achieve atraumatic, accurate osteotomy while preserving natural bone for grafting.

**Summary:**

Two female patients (ages 38 and 27) with symptomatic apical periodontitis of previously treated mandibular first molars were treated using this innovative approach. The methodology involved comprehensive clinical and radiographic examinations, including CBCT scans and digital oral scans. Virtual surgical planning was performed to design 3D‐printed surgical templates that predetermine osteotomy size and apical resection levels. The surgical procedure utilized (Altaqi kit) piezoelectric instruments specially designed for endodontic microsurgery for precise bone and root‐tip cutting and root‐end preparation under microscopic guidance. The key work in these cases was the ability to precisely and conservatively cut and reposition the bone window as an autogenous bone graft, secured with platelet‐rich fibrin plugs. This approach minimized surgical trauma by selectively cutting hard tissues while preserving soft tissues and reducing thermal damage. Both cases demonstrated successful clinical outcomes at 12–18‐month follow‐up, with patients remaining asymptomatic and showing routine healing without complications. The integration of piezoelectric technology with static surgical guides promoted early healing through bone‐preserving techniques. The study concludes that this combined approach offers significant advantages for endodontic microsurgery, particularly in complex anatomical scenarios near critical structures, despite preparation time and equipment costs.

## 1. Introduction

Surgical endodontics, specifically apicoectomy, is a procedure employed to save teeth with endodontic lesions unresponsive to conventional treatments. A crucial step in this procedure involves accurate osteotomy, lesion excision, and resection of the apical 3 mm of the root. This apical resection effectively removes over 90% of canal ramifications and lateral canals, thereby significantly reducing the likelihood of lesion recurrence [1, 2].

The traditional freehand surgery is still a valid approach in almost all endodontic surgical cases, which relies heavily on the surgeon′s ability to mentally map the anatomy from a cone‐beam computed tomography (CBCT) scan of the patient. Despite that, its application carries a high risk of damaging vital structures and/or removing excess bone [3–7].

To mitigate these shortcomings, digitally guided surgical endodontic approaches have been developed, demonstrating particular utility in complex anatomical scenarios [8–10]. The technological triad comprising guided implantology software, CBCT imaging, and 3D digital oral cavity scanning establishes the foundation for virtual planning in modern surgical endodontics [11–13]. Advancements in surgical precision through computer‐guided approaches (CGAs) have evolved, utilizing computer‐guided techniques categorized as either static or dynamic [14, 15]. The static guided surgery (SGS) approach involves creating a 3D‐printed surgical template or guide based on CBCT and optical scans. The guide directs the surgical instruments to ensure a precise osteotomy and apical resection. This method has been shown to improve accuracy and reduce surgical time [6, 14]. On the other hand, dynamic guided surgery (DGS) uses real‐time navigation, similar to GPS, to guide the surgeon′s handpiece without a physical template, thereby offering flexibility and accuracy [15, 16]. The osteotomy part can be performed using various burs and drills, including a surgical round bur, an implant drill, a trephine, and, most recently, ultrasonic (US) and piezoelectric (PIEZO) devices. The use of appropriate surgical tips for PIEZO and US in osteotomy offers several advantages for bone surgery, including precise, micrometric cuts with minimal invasiveness. It selectively cuts hard tissue while preserving soft tissues such as nerves and blood vessels, reduces thermal damage, and provides a nearly blood‐free surgical site, improving visibility and healing [17, 18].

The integration of a PIEZO device with a static surgical guide combines the benefits of both technologies. This synergy aims to enhance the accuracy and efficiency of bone window cutting, which is crucial for the success of the surgery [19]. In addition, it allows repositioning of the cut window in the bone to serve as an autogenous bone graft after the surgery [19].

This study includes two case reports demonstrating the benefits of combining advanced technologies (SGS with PIEZO) to perform atraumatic, accurate osteotomy while preserving natural bone as a graft.

## 2. Case Report

### 2.1. First Case

A 38‐year‐old female patient presented with pain, discomfort, and tenderness to percussion related to the lower left mandibular first molar. Clinical and radiographic examination revealed a previously root canal–treated (RCT) tooth with symptomatic apical periodontitis. On the periapical (PA) x‐ray and CBCT, there was a radiolucency, mainly related to the distal root encroaching on and almost perforating the lingual plate. The tooth was RCT twice and crowned, but no significant healing occurred over 2 years. The crown is both aesthetically and functionally acceptable to the patient, and she wants to keep it (Figure 1). The option of nonsurgical and microsurgical retreatment was presented to the patient, with all risks and benefits explained, and the patient decided to proceed with the microsurgical approach. The operator chose to perform a guided endodontic surgery using a 3D‐printed SGS and PIEZO for a precise, conservative, and atraumatic procedure.

**Figure 1:**
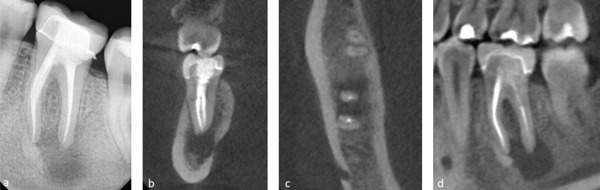
Preoperative radiographs. (a) PA x‐ray showed apical RL related mostly to D root. (b) CBCT coronal view showing the RL approaching the lingual plate and IAN canal. (c) CBCT axial view at the level of 2 mm of the apex showing the extension of the RL. (d) CBCT sagittal view showing the extension of the RL.

A CBCT scan was taken using the Planmeca Viso G5 unit (Planmeca, Finland) with the following acquisition settings: 90 kV tube voltage, 5–8 mA current, 17.5‐s exposure time, 0.300‐mm voxel resolution, and a 20 × 17 cm field of view (FOV). An intraoral digital scan was also obtained using the PANDA Smart A scanner (Suzhou Frequency Medical Technology Co. Ltd., China). The preoperative DICOM files from the CBCT exam, along with the STL surface scan data, were imported into Real Guide 5.0 (3DIEMME), a dedicated implant‐planning and surgical‐guide design software. On this platform, the PA lesion associated with the affected tooth was accurately identified and marked for treatment planning (Figure 2).

**Figure 2:**
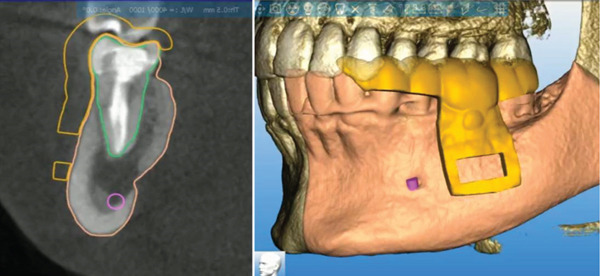
A snapshot of the planning software during the process of designing the 3D template.

In accordance with the evidence‐based principles governing contemporary endodontic microsurgery, virtual preoperative planning was employed to precisely define the osteotomy dimensions, bevel angulation, and the standardized 3‐mm apical root‐end resection depth, parameters collectively aimed at minimizing apical ramifications [4].

The virtually designed surgical templates were subsequently exported in STL format and transmitted to an Asiga Ultra 3D printer (Asiga, Australia) for physical fabrication. The surgical procedure was carried out under local anesthesia, administered as 4% articaine with epinephrine 1:100,000 (Septodont), with magnification provided by a Leica M530 operating microscope (Provido Dental, Milan, Italy). All surgical steps were executed in strict accordance with the established guidelines for contemporary endodontic microsurgery [4]. The accurate fit of the 3D‐printed surgical template was confirmed intraorally before commencing the procedure. A sulcular full‐thickness mucoperiosteal flap incorporating two vertical releasing incisions was then elevated, and the surgical template was seated into position. The osteotomy, designed as a bone window, was initiated using a PIEZO saw equipped with a Bone Cutter tip from the Altaqi kit (Woodpecker) (Figure 3a,b), showing the 3D guide and the PIEZO in place to start the osteotomy, and a bone window was cut according to the plan.

**Figure 3:**
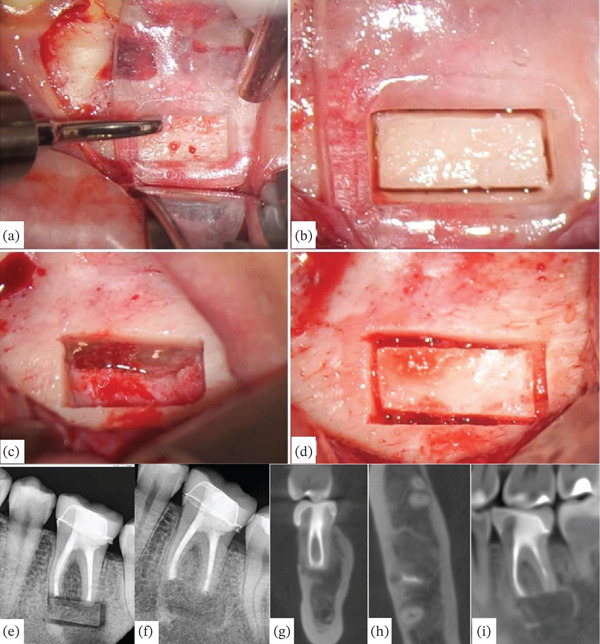
Clinical picture during the surgery. (a) The 3D guide and the PIEZO in place to start the osteotomy, (b) a bone window is cut according to the plan, (c) root‐end resection and lesion removed, (d) reposition of the bone window with PRF in place after the root‐end filling done, (e) immediate postoperative PA x‐ray showing the root‐end fill done and the cortical bone plate in place, (f) PA x‐ray showing complete healing after 18 months, (g) CBCT coronal view showing the complete resolution of the lesion and healing of the lingual cortical plate, and (h, i) CBCT axial and sagittal views, respectively, demonstrating the healing.

The elevated cortical plate was carefully lifted using a periosteal elevator, and the associated lesion was thoroughly removed with an excavator [20]. Root tip resection was carried out as planned using a PIEZO saw with a Root Cutter tip (Altaqi kit) (Figure 3c), demonstrating root‐end resection and removal of the lesion.

Lesion size was as follows: 13.2 mm width, 12.8 mm height, and 0.2 mm from IAN. Window size was as follows: 15 mm horizontal line, 5 mm vertical line, 3 mm within the root, and 2 mm below the apex of the root.

Root‐end preparation of both the mesial and distal roots was performed to a minimum depth of 3 mm into the root dentin, creating adequate cavities for retrograde filling placement, utilizing diamond‐coated PIEZO microsurgical retrotips (Shaper 3 and Shaper Left; Altaqi kit). Retrograde filling of both the mesial and distal roots was subsequently completed using EndoCem MTA (Maruchi, Korea).

Following completion and inspection of the root‐end fillings, with confirmation of adequate apical sealing, the cortical bone plate was repositioned at its original site [20]. Before fixation, a platelet‐rich fibrin (PRF) plug was placed in the socket to serve as a biological matrix and stabilize the cortical plate, preventing its displacement into the socket (Figure 3d).

Blood samples were collected following the PRF protocol [21] using a PC‐02 table centrifuge and collection kits supplied by Process (Nice, France). Briefly, about 10 mL of blood was drawn from the patient without anticoagulation into 10‐mL glass‐coated plastic tubes (WhFKLca, China) and immediately centrifuged at 3000 rpm for 12 min [21].

The full‐thickness flap was then repositioned and secured with sutures (PGA Suture 5/0; Omnia Spa, Fidenza, Italy). Postoperative PA radiography confirmed satisfactory root‐end filling placement and successful repositioning of the cortical plate (Figure 3e). The patient was prescribed a 5‐day postoperative regimen consisting of oral antibiotics (amoxicillin and clavulanic acid 1 g twice daily), analgesics (ibuprofen 400 mg three times daily), and chlorhexidine mouth rinse (0.2% three times daily), supplemented by both verbal and written postoperative instructions. Suture removal was performed at 7 days postoperatively. The postoperative course was uneventful, with no complications and a favorable healing trajectory.

#### 2.1.1. Recall

At the 18‐month postoperative follow‐up, the patient remained completely asymptomatic, with no tenderness to percussion, no bleeding on probing, and probing depths within clinically acceptable limits at all measured sites. PA and CBCT radiographic exams confirmed complete resolution of the previously identified lesion (Figure 3f–i), according to the Molven et al. [22] and Schloss et al. [23] criteria for success.

### 2.2. Second Case

A 27‐year‐old female patient presented with pain, discomfort, and tenderness to percussion related to the lower left mandibular first molar. Clinical and radiographic examination revealed a previously RCT tooth with symptomatic apical periodontitis. Preoperative PA and CBCT showed radiolucency involving the mesial and distal roots. The tooth was retreated and crowned 1 year ago, with no significant improvement in signs and symptoms. The crown is both aesthetically and functionally acceptable to the patient, and she wishes to keep it (Figure 4).

**Figure 4:**
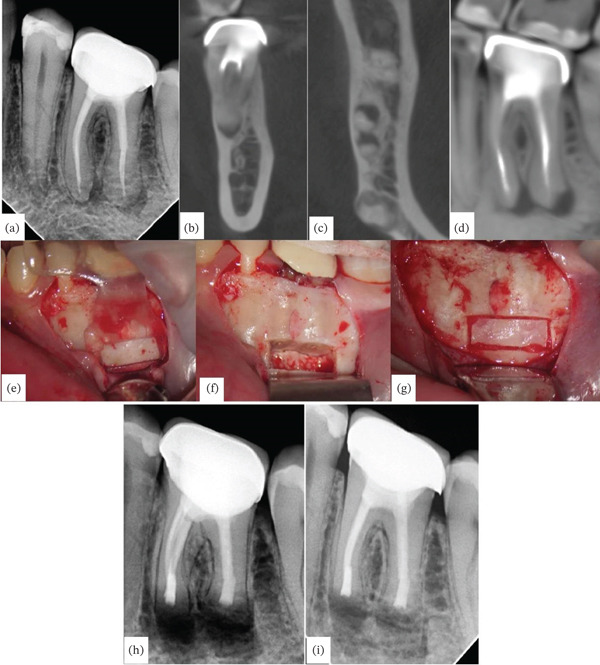
(a) PA x‐ray preoperative showing apical RL related to mesial and to distal roots; (b–d) CBCT coronal, axial, and sagittal views, respectively, showing slight apical RL associated with M and D roots; (e) intraoperative pictures of the surgery showing the 3D stent fixed in place; (f) the bone window cut, lesion cleaned and root‐end resection done; (g) reposition of the cortical plate in place over the PRF; (h) PA x‐ray showing immediately after the root‐end fill to check accuracy and before placing the PRF and bone cortical bone plate; and (i) postoperative with the bone plate in place.

The option of nonsurgical and microsurgical retreatment was presented to the patient, with all risks and benefits explained. The patient decided to proceed with the microsurgical approach. The operator chose to perform guided endodontic surgery using a 3D‐printed SGS and PIEZO for a precise, conservative, and atraumatic procedure. The 3D surgical guide was produced using the same protocol as in the first case. Surgery was carried out under local anesthesia (articaine 4% with 1:100,000, Septodont) using an operating microscope (Leica M530, Provido Dental, Milan, Italy) and performed in accordance with recommended guidelines for modern endodontic surgery [4].

Lesion size was as follows: 8 mm width for both roots together, 4 mm height, and 9 mm away from the IAN. Window size was as follows: 12 mm horizontal line, 5 mm vertical line, 3 mm within the roots, and 2 mm below the apex of the root.

The fit of the 3D‐printed surgical template was verified intraorally. A sulcular full‐thickness flap with two vertical releasing incisions was raised, and a template was placed [20]. The osteotomy (bone window) started with a PIEZO saw (Bone Cutter tip, Altaqi kit, Woodpecker). Elevation of the cortical plate was performed with a periosteal elevator, and the lesion was removed with an excavator. Resection of the root tips was performed as planned with a PIEZO saw (Root Cutter tip, Altaqi kit) (Figure 4e,f). Root‐end preparation of mesial and distal roots at least 3 mm into the root dentin to create a cavity for the retrograde fillings by using the diamond‐coated PIEZO microsurgical retrotips (Shaper 3, Shaper Left; Altaqi kit). Root‐end fillings of the mesial and distal roots were performed using EndoCem MTA (Maruchi, Korea).

The cortical plate was then repositioned to the site after a PRF plug (prepared as in the first case) was placed in the socket to serve as a matrix and prevent the plate from falling into the socket (Figure 4g). Before repositioning the cortical plate and after the root‐end fillings and inspection of the root‐end seals, a PA x‐ray was taken (Figure 4h). The full‐thickness flap was then repositioned and sutured (PGA Suture 5/0; Omnia Spa, Fidenza, Italy). The postsurgical intraoral PA x‐ray confirmed adequate root‐end fillings and repositioning of the cortical plate (Figure 4i). Oral antibiotics (1‐g amoxicillin and clavulanic acid, twice daily), analgesics (400‐mg ibuprofen, three times per day), and rinsing with chlorhexidine (0.2%, three times per day) were prescribed for 5 days, along with verbal and written postsurgical instructions. The sutures were removed 7 days postoperatively. No postoperative complications occurred, and the healing process was uneventful.

#### 2.2.1. Recall

At the 12‐month follow‐up, the patient was clinically asymptomatic, with no sensitivity to percussion, no bleeding on probing, and probing depths at all sites within normal limits. PA and CBCT radiographs showed significant healing and bone fill (Figure 5), according to the criteria of Molven et al. [22] and Schloss et al. [23] for success.

**Figure 5:**
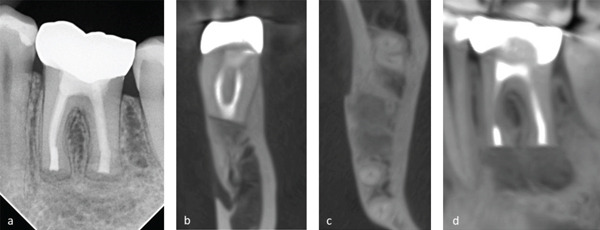
Twelve‐month recall radiographs. (a) PA x‐ray showing progressive healing and (b–d) CBCT coronal, axial, and sagittal views, respectively, demonstrating favorable healing in progress.

Both patients provided oral consent for the treatment and signed an informed consent form for the use of data and analysis in this report.

## 3. Discussion

Bone resorption of endodontic origin is a compelling indication for surgical intervention when chronic bacterial infection or pulp‐derived inflammation drives progressive, irreversible destruction of the root architecture or the surrounding alveolar bone, beyond the reach of nonsurgical RCT. Though RCT constitutes the cornerstone of endodontic management, surgery becomes indispensable in the presence of extraradicular infections, refractory apical lesions, or actively progressing perforating resorption and pathological entities that demand direct surgical access, precise visual identification, and thorough debridement of compromised tissues to achieve predictable healing outcomes [1, 4].

The dimensions of bony defects significantly impact patient outcomes and postendodontic healing. Extensive bone loss can lead to delayed or unsuccessful healing and an increased risk of postoperative complications [24, 25]. The height of the remaining buccal bone plate is also a crucial factor in healing outcomes, with insufficient marginal bone, often due to unintended osteotomy extension, reducing surgical success rates [25]. Excessive osteotomy can inadvertently occur, especially in the molar region with apical lesions on distal roots, due to access challenges [26].

The 3D template procedure guide has been introduced for orthograde endodontic access cavity preparation and root canal location [9, 27], and it has recently been widely used for EMS [3]. Surgical templates are particularly beneficial in complex cases near critical anatomical structures, such as adjacent roots, the inferior alveolar nerve, the mental foramen, and the maxillary sinus, which can impede access to the root apex [26]. So, guided surgery utilizing customized surgical templates can help navigate these critical landmarks, thereby expanding the applicability of endodontic microsurgery [19].

In this case, a static 3D‐printed surgical template was employed for guided, minimal osteotomy to create a surgical window over the apical lesions. This approach minimized surgical trauma, especially with the help of PIEZO. The harvested cortical bone was then repositioned as an autogenous bone graft to promote early healing. Postoperative pain and swelling are common complications in endodontic surgery [28, 29]. Reducing osteotomy extent through techniques like the “guided window” osteotomy with a 3D‐printed template and PIEZO can minimize these complications and enhance healing [19].

PIEZO instruments offer precise and safe cutting of hard tissues while preserving surrounding soft tissues. Their “air–water cavitation effect” also improves surgical field visibility by creating a clean, blood‐free environment [4].

Our observations in the present cases support the American Association of Endodontists′ 2025 publication on the use of Piezotome, which has revolutionized apical endodontic surgery by offering a precise, minimally invasive alternative to conventional instruments. Its ability to selectively remove bone while minimizing tissue trauma, heat generation, and patient discomfort highlights its value in modern endodontic practice. By improving apical access, preserving bone integrity, and promoting faster recovery, it has significantly enhanced both clinical outcomes and patient satisfaction [30].

PRF is an autologous blood‐derived biomaterial that, when placed in extraction sockets or surgical sites, releases growth factors to accelerate healing, prevent complications, and enhance bone and soft‐tissue regeneration in various dental procedures [31]. In the present cases, PRF was used as a filler material instead of a bone graft and to hold the bone plate from falling into the surgical site. It has been documented to be effective in optimizing postsurgical outcomes, specifically by enhancing soft‐tissue healing, reducing the incidence of alveolar osteitis, and decreasing postoperative pain [32].

The incorporation of a membrane into guided bone regeneration (GBR) is generally considered beneficial, as it provides mechanical stability and acts as a barrier against infiltration of surrounding soft‐tissue cells into the grafted site [33].

Existing evidence presents conflicting findings regarding the role of membrane use in determining GBR outcomes, suggesting that the procedure′s success is multifactorial [34–37]. In the present cases, the autogenous bone plate served as a natural hard‐tissue barrier, resulting in favorable outcomes and uneventful healing.

The bone window technique, using PIEZO and PRF, employed in the present cases, provided adequate stability of the repositioned bony window over the surgical site. Periosteal integrity was preserved through precise incision technique and gentle flap management, allowing tension‐free primary closure, consistent with a previous study by U. Kim et al., who introduced this concept [19].

It is noteworthy that the original protocol described by U. Kim et al. [19] did not incorporate PRF; instead, a particulate bone graft was placed at the surgical site, followed by repositioning of the bone plate and coverage with a resorbable membrane. Furthermore, the PIEZO surgical tips used in the present study were sourced from the Altaqi kit, which was specifically engineered for endodontic microsurgery, in contrast to standard PIEZO surgery tips, which are wider in diameter and less conservative. The Altaqi kit features a dedicated bone window cutter, a root‐end cutting tip, and a retrograde preparation tip, all designed to ensure a highly precise, conservative approach—a notable refinement over the instrumentation used in the original technique.

Despite significant advancements and favorable outcomes, these advanced technologies—CBCT, optical scanning, 3D‐printed surgical guides, and piezo surgery—have several limitations that must be addressed. These include additional time and costs for guide preparation, a substantial investment in equipment, and higher service fees for the patient compared to conventional freehand endodontic surgery. The outcomes of the presented cases emphasize the use of advanced technologies to enhance patient well‐being by minimizing surgical trauma and time and increasing the accuracy of surgical procedures.

## 4. Conclusions

The combination of a PIEZO device and a static surgical guide appeared to offer several practical advantages in endodontic microsurgery, as observed in the cases presented. This approach seemed to improve accuracy during bone window preparation, reduce surgical trauma, and allow for the preservation and repositioning of the bone window as an autogenous graft, which may have supported early healing. The authors suggest that such a combined technique may benefit patients by potentially minimizing trauma while enhancing procedural precision.

## Author Contributions

Mohammed Mashyakhy: conceptualization (lead), methodology (equal), writing—original draft preparation (lead), and writing—review and editing (lead). Zaher Altaqi: methodology (equal), designing the surgical guide (lead), and performing the surgery (lead).

## Funding

No funding was received for this manuscript.

## Disclosure

All procedures were conducted independently, and the kit was utilized solely as a tool for the surgical workflow described herein. All authors have read and approved the final version of the manuscript. The corresponding author had full access to all data in this study and takes full responsibility for the integrity of the data and the accuracy of the analysis.

## Ethics Statement

The patient provided oral consent for the treatment and signed an informed consent form for the use of data and analysis in this report.

## Conflicts of Interest

The authors declare no conflicts of interest.

## Data Availability

The data that support the findings of this study are available upon request from the corresponding author. The data are not publicly available due to privacy or ethical restrictions.
